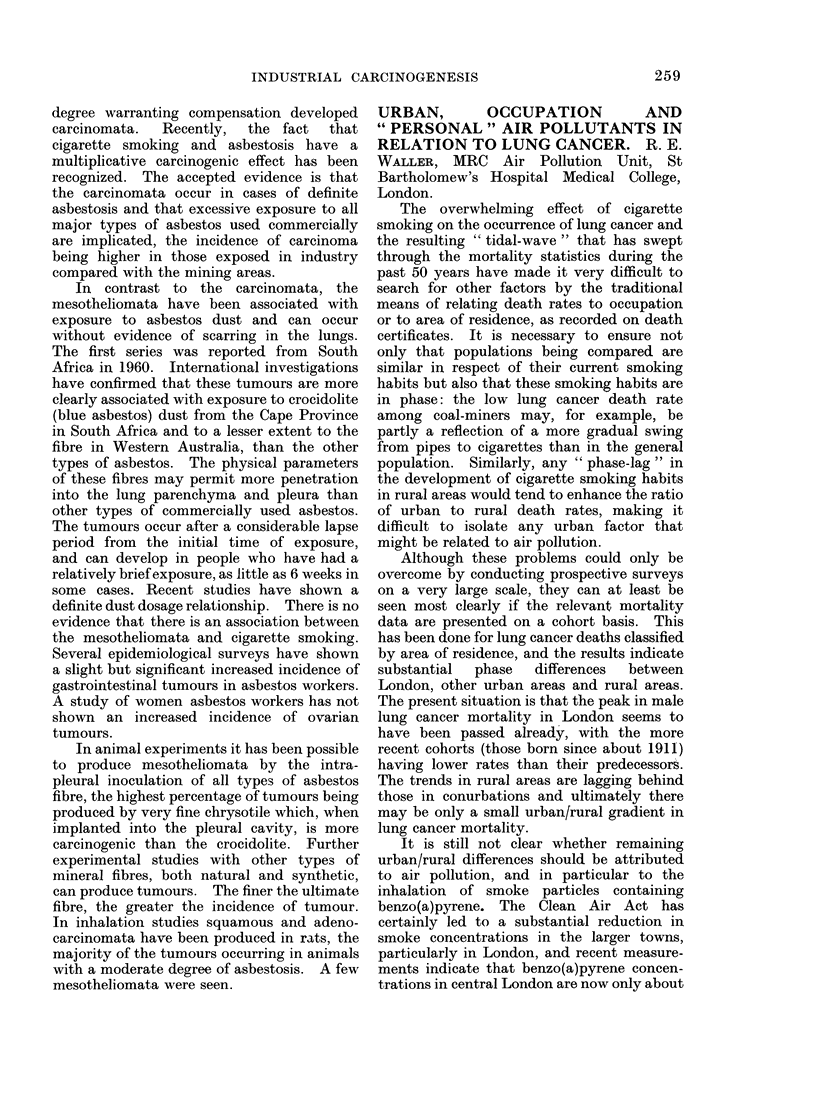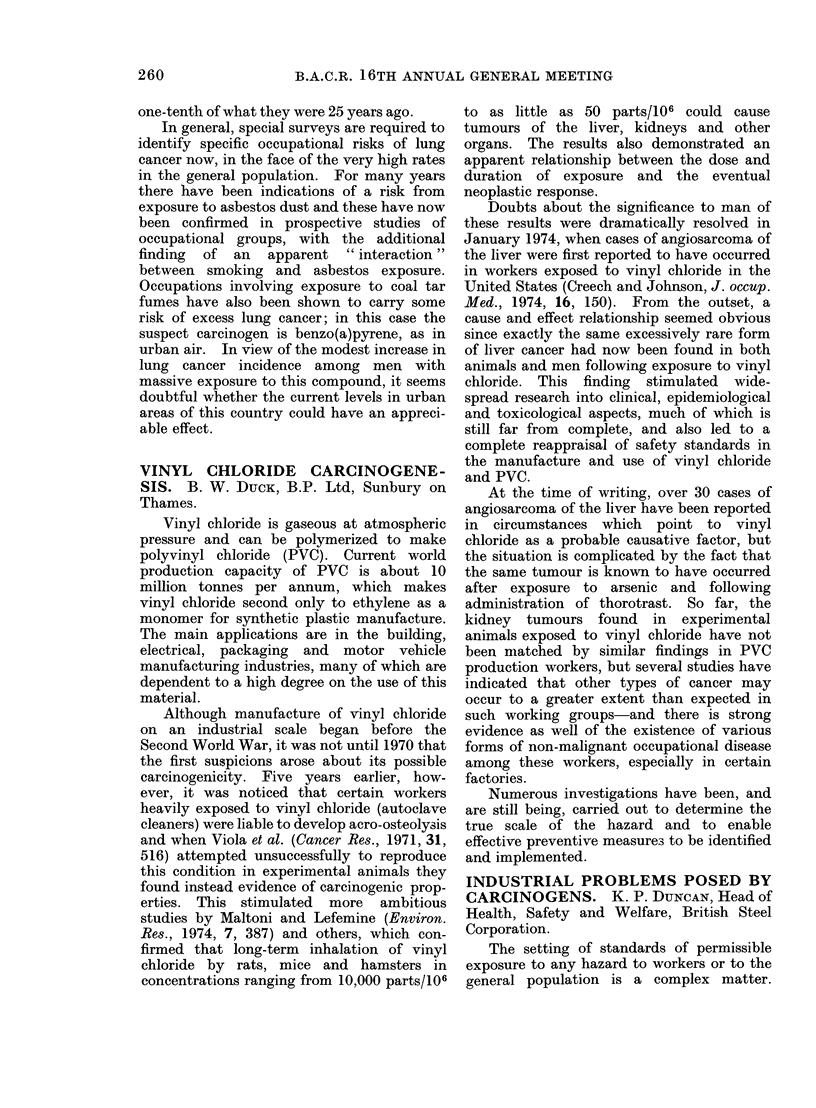# Proceedings: Urban, occupation and "personal" air pollutants in relation to lung cancer.

**DOI:** 10.1038/bjc.1975.207

**Published:** 1975-08

**Authors:** R. E. Waller


**URBAN, OCCUPATION AND " PERSONAL " AIR POLLUTANTS IN RELATION TO LUNG CANCER.** R. E. Waller, MRC Air Pollution Unit, St Bartholomew's Hospital Medical College, London.

The overwhelming effect of cigarette smoking on the occurrence of lung cancer and the resulting " tidal-wave " that has swept through the mortality statistics during the past 50 years have made it very difficult to search for other factors by the traditional means of relating death rates to occupation or to area of residence, as recorded on death certificates. It is necessary to ensure not only that populations being compared are similar in respect of their current smoking habits but also that these smoking habits are in phase: the low lung cancer death rate among coal-miners may, for example, be partly a reflection of a more gradual swing from pipes to cigarettes than in the general population. Similarly, any " phase-lag " in the development of cigarette smoking habits in rural areas would tend to enhance the ratio of urban to rural death rates, making it difficult to isolate any urban factor that might be related to air pollution.

Although these problems could only be overcome by conducting prospective surveys on a very large scale, they can at least be seen most clearly if the relevant mortality data are presented on a cohort basis. This has been done for lung cancer deaths classified by area of residence, and the results indicate substantial phase differences between London, other urban areas and rural areas. The present situation is that the peak in male lung cancer mortality in London seems to have been passed already, with the more recent cohorts (those born since about 1911) having lower rates than their predecessors. The trends in rural areas are lagging behind those in conurbations and ultimately there may be only a small urban/rural gradient in lung cancer mortality.

It is still not clear whether remaining urban/rural differences should be attributed to air pollution, and in particular to the inhalation of smoke particles containing benzo(a)pyrene. The Clean Air Act has certainly led to a substantial reduction in smoke concentrations in the larger towns, particularly in London, and recent measurements indicate that benzo(a)pyrene concentrations in central London are now only about one-tenth of what they were 25 years ago.

In general, special surveys are required to identify specific occupational risks of lung cancer now, in the face of the very high rates in the general population. For many years there have been indications of a risk from exposure to asbestos dust and these have now been confirmed in prospective studies of occupational groups, with the additional finding of an apparent "interaction" between smoking and asbestos exposure. Occupations involving exposure to coal tar fumes have also been shown to carry some risk of excess lung cancer; in this case the suspect carcinogen is benzo(a)pyrene, as in urban air. In view of the modest increase in lung cancer incidence among men with massive exposure to this compound, it seems doubtful whether the current levels in urban areas of this country could have an appreciable effect.